# Widespread *pfhrp2/3* deletions and HRP2-based false-negative results in southern Ethiopia

**DOI:** 10.1186/s12936-024-04904-3

**Published:** 2024-04-17

**Authors:** Bacha Mekonen, Sisay Dugassa, Sindew Mekasha Feleke, Boja Dufera, Bedasa Gidisa, Aderaw Adamu, Aynalem Mandefro, Geremew Tasew, Lemu Golassa

**Affiliations:** 1 Aklilu Lemma Institute of Pathobiology, Addis Ababa University, Addis Ababa, Ethiopia; 2 Malaria and NTDs Research Team, Bacterial, Parasitic, and Zoonotic Diseases Research Directorate, Ethiopian Public Health Institute, Addis Ababa, Ethiopia; 3 Malaria and NTDs Research Team, Armeur Hansen Research Institute, Addis Ababa, Ethiopia; 4 Department of Medical Laboratory Science, College of Medicine and Health Science, Wollo University, Dessie, Ethiopia

**Keywords:** *Plasmodium falciparum*, pfhrp2/3, Gene deletions, Health centers, PfHRP2-RDT, Southern Ethiopia

## Abstract

**Background:**

Rapid diagnostic tests (RDTs) play a significant role in expanding case management in peripheral healthcare systems. Histidine-rich protein-2 (HRP2) antigen detection RDTs are predominantly used to diagnose *Plasmodium falciparum* infection. However, the evolution and spread of *P. falciparum* parasite strains with deleted *hrp2/3* genes, causing false-negative results, have been reported. This study assessed the diagnostic performance of HRP2-detecting RDTs for *P. falciparum* cases and the prevalence of *pfhrp2/3* deletions among symptomatic patients seeking malaria diagnosis at selected health facilities in southern Ethiopia.

**Methods:**

A multi-health facilities-based cross-sectional study was conducted on self-presenting febrile patients seeking treatment in southern Ethiopia from July to September 2022. A purposive sampling strategy was used to enroll patients with microscopically confirmed *P. falciparum* infections. A capillary blood sample was obtained to prepare a blood film for microscopy and a RDT using the SD Bioline™ Malaria Pf/Pv Test. Dried blood spot samples were collected for further molecular analysis. DNA was extracted using gene aid kits and amplification was performed using nested PCR assay. *Exon 2* of *hrp2* and *hrp3*, which are the main protein-coding regions, was used to confirm its deletion. The diagnostic performance of RDT was evaluated using PCR as the gold standard test for *P. falciparum* infections.

**Results:**

Of 279 *P. falciparum* PCR-confirmed samples, 249 (89.2%) had successful *msp-2* amplification, which was then genotyped for *hrp2/3* gene deletions. The study revealed that *pfhrp2/3* deletions were common in all health centres, and it was estimated that 144 patients (57.8%) across all health facilities had *pfhrp2/3* deletions, leading to false-negative PfHRP2 RDT results. Deletions spanning exon 2 of *hrp2*, exon 2 *of hrp3*, and double deletions (hrp2/3) accounted for 68 (27.3%), 76 (30.5%), and 33 (13.2%) of cases, respectively. The study findings revealed the prevalence of *P. falciparum* parasites lacking a single *pfhrp2-/3-*gene and that both genes varied across the study sites. This study also showed that the sensitivity of the SD Bioline PfHRP2-RDT test was 76.5% when PCR was used as the reference test.

**Conclusion:**

This study confirmed the existence of widespread *pfhrp2/3-* gene deletions, and their magnitude exceeded the WHO-recommended threshold (> 5%). False-negative RDT results resulting from deletions in *Pfhrp2/3*- affect a country’s attempts at malaria control and elimination. Therefore, the adoption of non-HRP2-based RDTs as an alternative measure is required to avoid the consequences associated with the continued use of HRP-2-based RDTs, in the study area in particular and in Ethiopia in general.

## Background

Malaria is a life-threatening vector-borne disease caused by *Plasmodium* parasite species. Malaria is endemic in 88 countries, affecting approximately half of the world’s population, with the highest burden in sub-Saharan Africa. An estimated 95% of all malaria cases and deaths occur in Africa [1]. Ethiopia is one of the most malaria-endemic countries in the world, with an estimated 68% of the population and 75% of the country’s geographic area being at risk. The economic burden of infections in the country is disproportionately high because of the overlap between the peak transmission seasons and major agricultural activities in rural areas. In Ethiopia, the magnitude of malaria distribution varies in time and space and is characterized by its locality, seasonality, and unstable transmission forms. The country is home to both *Plasmodium falciparum* and *Plasmodium vivax,* with *Anopheles arabiensis* as the principal mosquito vector [2, 3].

Despite collective efforts that have resulted in malaria reduction, it continues to be a pressing global health issue, particularly in tropical and subtropical regions Among other human malaria-causing parasites, *P. falciparum* causes the most severe infections and is responsible for the majority of mortality and morbidity [4].

Accurate early diagnosis and prompt treatment are the key strategies for controlling and preventing malaria in Ethiopia. Indeed, microscopy and rapid diagnostic tests (RDTs) are widely used malaria diagnostic tools in clinical settings. Although microscopy is the gold standard diagnostic method, it is not always feasible in all settings, as it requires expertise, time, and infrastructure [5]. The development of malaria RDTs has contributed to case management, detection, and surveillance and has become one of the most effective diagnostic products in global health [6, 7]. Globally, 3.1 billion RDTs were sold in 10 consecutive years (2010–20), with 81% of these in countries of sub-Saharan Africa (SSA). In Ethiopia, RDTs were introduced in late 2004 and continue to improve case management, especially in peripheral health facilities/health posts, where most cases (> 70%) are found [8, 9].

RDTs are antigen detection tests, using antibodies specific for antigens found in *Plasmodium* species, including lactose dehydrogenase (pLDH), aldolase, and histidine-rich protein2 (HRP2*)*. Among these, HRP2-based RDTs are the most effective for detecting *P. falciparum*, the most common malarial parasite [8, 10]. Compared with other commercially available RDTs, they are also more sensitive and heat-stable. HRP2-based RDTs detect not only HRP2 but also its isoform, HRP3, which has high sequence similarity to HRP2 [10].

Despite their advantages, RDTs are under serious threat because of the emergence of parasites not or little expressing the target proteins due to mutations in the genes that encode them [11–16]. This has been reported in both laboratory and field isolates [17, 18]. In recent years, studies have shown that a large number of *P. falciparum* cases have been reported to be negative by HRP2 detection RDTs, raising concerns regarding its sensitivity. *Pfhrp2/3* gene deletions have resulted in false negative results of HRP2-based RDTs [19], in particular, in the Horn of Africa region [20].

*Plasmodium falciparum* parasites lacking histidine-rich protein 2/3 have been reported in Ethiopia with different frequencies of gene deletions from different parts of the country. For instance, studies conducted in 2021–2022 [21–23] reported a significant number of *P. falciparum* cases that were missed by HRP2-RDTs due to *pfhrp2/3* gene deletions. According to the World Health Organization (WHO) guidelines, an *hrp2* deletion prevalence exceeding 5% calls for a switch from HRP2-based RDTs to an alternative test [19]. Unfortunately, non-HRP2-based RDTs are less common, less sensitive, and more susceptible to heat and humidity [24]. Adequate evidence for the presence and magnitude of *P. falciparum* parasites with *pfhrp2/3* gene deletions that cause false-negative PfHRP2-RDTs is important for policy decisions [25]. Therefore, this study aimed to assess the diagnostic performance of HRP2-based RDTs for *P. falciparum* infections and the prevalence of *pfhrp2/3* gene deletions among symptomatic patients seeking malaria diagnosis in selected health facilities in southern Ethiopia.

## Methods

### Study area

The study area is located in the southern and southwestern parts of Ethiopia, with an estimated 20.5 million people belonging to diverse ethnic groups. Approximately 91.1% of the population lives in rural areas [26]. The main sources of local livelihood are agriculture and livestock herding. The region is endemic to malaria, with different transmission intensities. Notably, *P. falciparum* is the primary malaria species, accounting for over 60% of all cases [27].

**Fig. 1:**
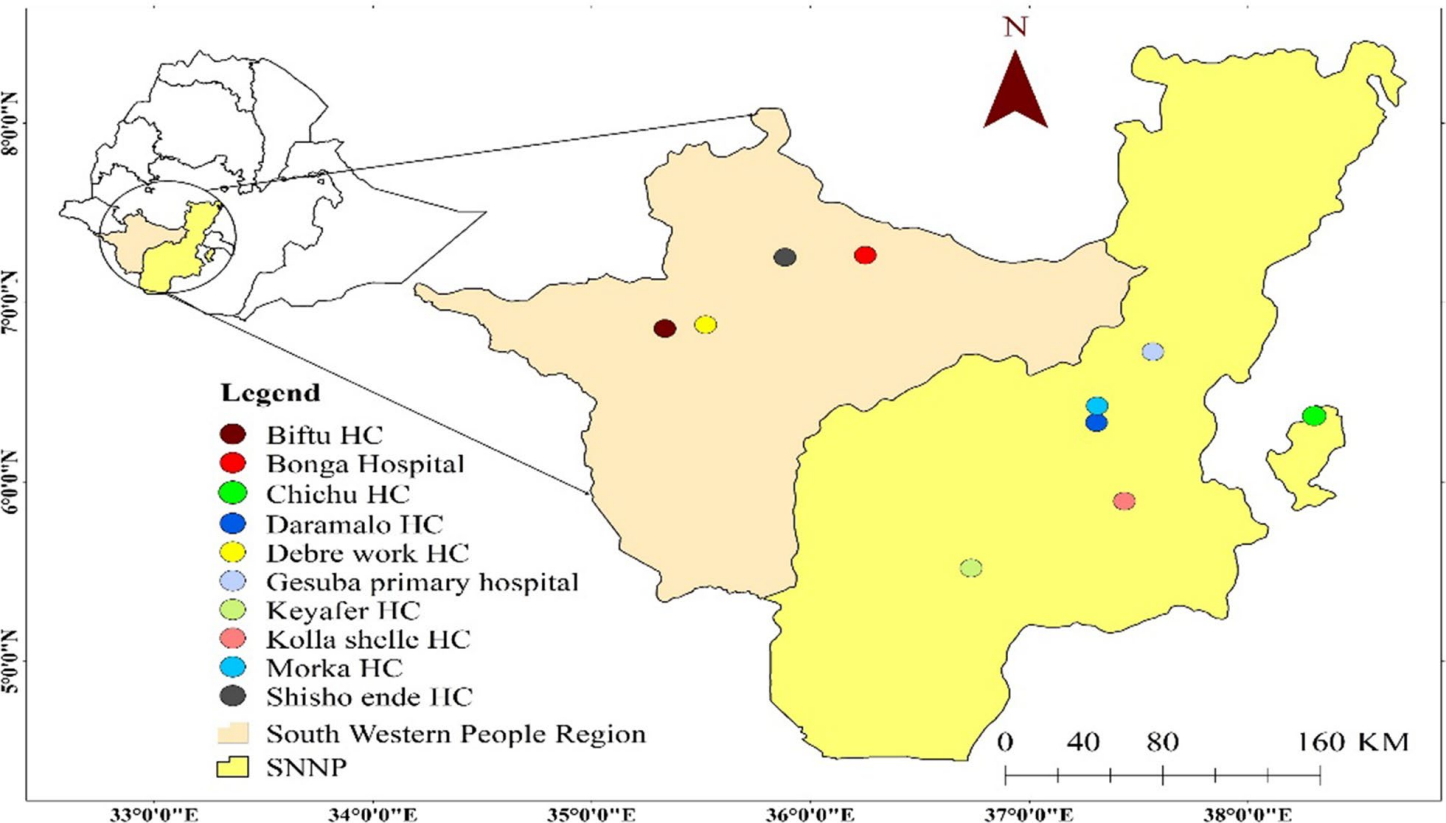
Map of Ethiopia showing study area locations in southern Ethiopia in 2023 (SNNP: South Nations, nationalities, and people; HC: Health Centre)

The study area for this research comprises several districts with diverse ecological and epidemiological areas located in these regions. The study covered seven zonal areas of the regions; Kaffa (Bonga Hospital and Shishonde Health Centre), Bench Sheko (Biftu and Debrewerk Health Centre), Gedio (Chichu Health Centre), South Omo (Keyafer Health Centre), Gamo (Kolashelle Health Centre), Gofa (Morka and Daramallo Heath Centre), and Wolaita (Gesuba Primary Hospital) zones are the study locations and areas. The study area map was created using ArcMap software (version 10.7.1) (Fig. 1).

### Study design and period

This multi-health facility-based cross-sectional study was conducted among ten public health facilities in southern Ethiopia (Fig. 1). The study areas were selected based on simple random sampling, considering the geographical representation and distribution of malaria transmission across regions. The study was conducted between July and September 2022, during the rainy season.

### Sample size determination

The sample size was determined using the following equation:

Sample size **n = **$n\ge deft\left[\frac{{Z}^{2}(P)(1-P)}{{d}^{2}}\right]$, where **n** is the required sample size*,* **Z** = 95% statistic for the level of confidence limits*,* **P** = previous *pfhrp2*-deletions prevalence*,* **d** = margin of error*, ****deft*** = sampling design effect = 2, and the probability of committing a type-1 error = 95% [19]. Considering the estimated previous prevalence (*pfhrp2* gene deletions), P = 9.7% (d = 0.05) and a 95% confidence level (z = 1.96).

Initially, this calculation indicated a need for a minimum of 270 participants. However, considering the WHO master protocol for gene deletion procedures [24], adjustment was made to include enrollment target. Consequently, a total of 370 individuals infected with *P. falciparum* from ten health facilities located in southwestern Ethiopia were recruited (Fig. 1), averaging between 37 and 40 confirmed cases from each facility through microscopy.

### Study procedures and patient enrollments

This study was conducted in a clinical setting. Patients who were febrile at the time of enrollment, or who had a history of fever within the past 24 h and who visited the closest public health centres to seek treatment were screened. Participants with microscopically confirmed *P. falciparum* results were enrolled using a purposive sampling strategy. A specific identification barcode was provided for each participant. All consent and assent were obtained in written form. Patients with complicated malaria were excluded from this study. The confirmed cases were treated according to the national treatment guidelines. Structured questionnaires were used to collect sociodemographic information, clinical features, and laboratory results for each participant.

A tally was used to enroll participants from each study site. Those microscopically confirmed as *P. falciparum* were tested by one step SD Bioline™ malaria Pf/Pv, which targets HRP2 for *P. falciparum* and LDH for *Plasmodium vivax* (Product code 05FK80, LOT 05DDG005B; Standard Diagnostics, Inc., Republic of Korea), and the results were recorded carefully. Discordant results were considered when the test results were positive for *P. falciparum* on microscopy and negative on RDTs. In addition, a microscopically mixed infections were considered as discordant when it became *P.v-*positive and *P.f-*negative on RDTs.

Participants with microscopically confirmed *P. falciparum* results were enrolled using a purposive sampling strategy. Standard operating procedures were prepared for each study site in both the local language (Amharic) and English versions for reference. On-the-job training was provided to the laboratory technicians at each study site. Strict follow-up and supervision were performed.

### Laboratory procedures

#### Microscopy

For microscopic procedures, thin and thick blood films were prepared from the finger-prick blood samples. Blood smears were stained with 10% Giemsa for 10 min and examined through a 100× oil immersion objective. For thick smears, as per WHO recommendations, parasite density was measured against 200 white blood cells (WBCs), assuming a mean WBC count of 8000/μL [28]. The slides were read by two independent microscopists; if there were any discrepancies between them, especially in the slide positivity or species differentiation, a third blind microscopist was added. Additionally, the Obare method calculation was performed if the two microscopists’ quantification of the parasite differed, and a third blind reader quantified if necessary.

#### Rapid diagnostic tests (RDTs)

The SD Bioline™ Malaria Pf/Pv RDT was performed according to the manufacturer’s instructions as indicated in the insert kit.

#### Dried blood spot (DBS) collections

A minimum of three blood spots were collected using a finger prick on protein saver cards (Whatman 903TM LOT 7224921 W201 Global Life Science Solutions Operations Ltd, Amersham UK) from eligible study participants. The collected DBSs were dried, labelled with particular barcodes, and packed with desiccants in zip-lock bags according to standard procedures and laboratory guidelines separately provided for each site. The desiccated blood specimens were transported in a temperature-controlled container to the Medical Parasitology Laboratory at ALIPB, AAU, where they were subsequently preserved at − 20 °C in Addis Ababa, Ethiopia.

#### Molecular analysis

Molecular analysis was conducted at the Akililu Lemma Institute of Pathobiology, Addis Ababa University, and all molecular laboratory analyses nucleic acid amplification tests (NAAT). The laboratory has excellent PCR performance and has been registered with the UK National External Quality Assessment Service (NEQAS) since 2021.

#### DNA extraction procedures

Briefly, DBS samples were arranged in the laboratory with a new identification number (Lab. ID). A 6-mm DBS punch was punched out and placed into a 1.5-mL Eppendorf tube. Between each sample punch, the puncher was disinfected with clean plain paper. Genomic DNA was extracted using an extraction kit known as the Geneius™ Micro GDNA kit (Geneaid Biotech Ltd, Taiwan). To avoid cross-contamination, working and stock samples were separated into centrifuge tubes. Extracted DNA was kept at − 20 °C until used for amplification.

### Genus-specific (18S ribosomal RNA) and targeted genotyping for *P. falciparum*

A nested PCR-based approach was used for two-round amplification. The first round of PCR (N1) confirmed the presence of *Plasmodium* parasites, and the second round (N2) was used to differentiate *P. falciparum* species.

The first PCR employed the genus-specific oligonucleotide primer pairs rPLU5 and rPLU6 and the N2 species-specific oligonucleotide primer pairs rFAL1 and rFAL2 (Table 1), as previously described [29]. Briefly, the N1 program and cycling conditions were as follows: initial denaturation at 95 °C for 10 min; 35 cycles of denaturation at 95 °C for 60 s, annealing at 58 °C for 60 s, and extension at 72 °C for 90 s; and a final extension at 72 °C for 10 min. The second round of PCR was conducted at 95 °C for 10 min, followed by denaturation at 95 °C for 60 s, annealing at 58 °C for 60 s, extension at 72 °C for 90 s, and a final extension at 70 °C for 10 min. A total of 30× PCR cycles were performed (Table 1).

**Table 1:** Primer assays used for genus- and *P. falciparum* species-specific amplification

	Name of primer	Sequences 5′–3′	Expected band size (bp)
N1 (genus-specific)	rPLU6	TTAAAATTGTTGCAGTTAAAACG	1200
	rPLU5	CCT GTT GTT GCC TTA AAC TTC	
N2 (species-specific)	rFAL1	TTA AAC TGG TTT GGG AAA ACC AAA TAT ATT	205
	rFAL2	ACA CAA TAG ACT CAA TCA TGA CTA CCC GTC	

Amplicons were determined by gel electrophoresis on 1.5% agarose stained with ethidium bromide, which was run for approximately one hour at 120 V. The migration of PCR products in the gel was visualized using a Benchtop 2UV transilluminator machine and photographed. A 100 (BioChain 100-bp DNA Ladder) DNA ladder was used to estimate the movement of the base pairs. Specific positive 3D7 and nuclease-free negative water controls were used to monitor the quality of the protocol.

#### Merozoite surface protein 2 genotyping

Merozoite surface protein 2 (MSP-2) genotyping was conducted primarily to assess the quality of the extracted parasite DNA, specifically aiming to validate its integrity and avoid potential misclassification of gene deletions attributed to DNA quality issues. For *msp-2* genotyping, N1 and N2 rounds of PCR were run in 20 PCR strip volumes with 0.5 µL of each forward and reverse primer. For N1 amplification, the primer sets forward (F1)—5′-GAA GGT AAT TAA AAC ATT GTC 3′ and reverse (R1)-5′-GAT GCT GCT CCA CAG-3′ were utilized. For N2 amplification, the primer sets forward (F2) 3′-GAG TAT AAG GAG AAG TAT and reverse (R2)-5′-CTA GAA CCA TGA ATA TGT CC-3′ were employed. N1 PCR amplification was conducted with an initial denaturation at 95 °C for 3 min, followed by 38 cycles at 95 °C for 60 s, 58 °C for 60 s, 72 °C for 90 s, and 72 °C for 60 s for the final extension, as adopted from [29]. The amplified N2 PCR products were separated by 1.5% agarose gel electrophoresis at 120 V for 1 h.

### Genotyping of *pfhrp2* and *pfhrp3*

Molecular analysis of *pfhrp2* and *pfhrp3* was conducted by amplifying the main coding region segment, *exon-2,* as previously described [30]. Samples were tested for successful amplification of *msp-2* before genotyping *pfhrp2* and *pfhrp3* genes, and those with positive *msp2* gene amplifications were tested for *hrp2* and *hrp3* genes. Briefly, a total PCR reaction mixture volume of 25 μL was made of 12.5 µL of 2× Go Taq hot start green master mix, 1 μL each of forward and reverse primers, 3 μL of template DNA, and 7.5 µL of molecular grade water were used to amplify *hrp2*/3 exon2 gene. Primer sequences employed for the amplification of hrp2-exon-2 were as follows: the forward primer (F) had the sequence 5′-AAG GAC TTA ATT TAA ATA AGA-3′, while the reverse primer (R) had the sequence 5′-AAT AAA TTT AAT GGC GTA GGC A-3′. In the case of hrp3-exon-2 amplification, the forward primer (F) used had the sequence 5′-AAA GCA AAA GGA CTT AAT TC-3′, and the reverse primer (R) had the sequence 5′-TGG TGT AAG TGA TGC GTA GT-3′ [21, 31, 32]. A single round of 40 amplification cycles was performed under the following cycling conditions: 94 °C for 10 min, followed by 94 °C for 50 s, 55 °C for 50 s, and 70 °C for 1 min. Reference strains of DNA from Dd2, HB3, and 3D7 were used as controls for *hrp*2 deletion, *hrp*3 deletion, and absence of deletion, respectively. After being stained with ethidium bromide, the amplified segments of the *hrp2* and *hrp3* genes were separated by electrophoresis on a 2% agarose gel. A 100 bp DNA ladder was used to see the DNA bands and determine the expected amplicon size.

### Data analysis

All field and laboratory procedures were performed according to the standard operating procedures. The collected field data were double-checked for consistency and completeness. The data were then entered into Epi Info software (version 7.2.5.0), exported to an Excel sheet, and analyzed using SPSS version 20.0 (SPSS, Chicago, IL, USA). Frequency and percentage calculations were made using descriptive statistics. When evaluating the RDTs’ sensitivity, specificity, accuracy, and negative and positive predictive values PCR analysis was used as the gold standard test. The level of agreement between the diagnostic tools was determined using Cohen’s kappa coefficient. Moreover, the Chi-Square test was performed to evaluate the relationship between the explanatory variables and determine significance. All results were calculated and presented at 95% confidence intervals (CI), and a p-value of ≤ 0.05 was used to determine if a result was significant.

## Results

### Sociodemographic and clinical features of the study participants

A total of 3510 self-reported febrile individuals were screened for malaria at ten health centres to enroll 370 microscopically confirmed *P. falciparum*-positive patients. Of 370 patients, seven were excluded from the study due to incomplete data and under-quality DBS, leaving 363 study participants who completed the study. From 363 study participants, 54.8% (199/363) and 45.2% (164/363) were male and female, respectively. The largest age group was 16 to 30 years (43.8%), followed by those aged 6–15 years (25.3%). The majority of participants (63%) lived in rural areas (Table 2A).

**Table 2:** Study participants (N = 363): baseline sociodemographic and clinical characteristics selected for molecular analysis in Southern Ethiopia in 2023

Study variables	Category	n	(%)	
A: Sociodemographic characteristics of study participants, n = 363 (100%)				
Sex	Male	199	54.8	
	Female	164	45.1	
Age strata in years	≤ 5	34	9.9	
	6 to 15	87	23.9	
	16 to 30	164	45.1	
	31 to 50	73	20.1	
	≥ 51	5	1.3	
House locations	Urban	134	37	
	Rural	229	63	
B: Clinical characteristics of study participants, n = 363 (100%)				
Previous malaria exposures in the past months	No	158	43.5	< 0.001
	Yes	205	56.4	
Parasite destiny p/µL blood	< 5000	178	49	< 0.001
	> 5000	185	51	
Fever within the past 24 h	No	28	7.7	0.0082
	Yes	335	92.28	
Current fever	No	90	24.8	< 0.001
	Yes	273	75.2	
Headache	No	39	10.7	0.001
	Yes	324	89.25	
Joint pain	No	71	19.5	0.001
	Yes	292	80.5	
Nausea and vomiting	No	178	49	0.703
	Yes	185	51	
Feeling cold	No	183	50.7	0.78
	Yes	180	49.3	

Of the 363 participants, 56.4% (205) reported a history of malaria exposure over the past months, and the majority of participants (77.2%) had a parasite density of less than 5000 per microlitre of blood. The majority of participants had a reported fever in the past 24 h (92.28%), and 75.2% had a current fever (at the time of examination). Joint pain was reported by 80.5% of the participants, followed by headache (89.25%). Feeling cold was reported by almost half of the participants (49.3%), whereas the frequencies of nausea and vomiting were almost equal (51%) (Table 2B).

### Molecular analysis

#### Genus- and species-specific amplification and detection

**Fig. 2:**
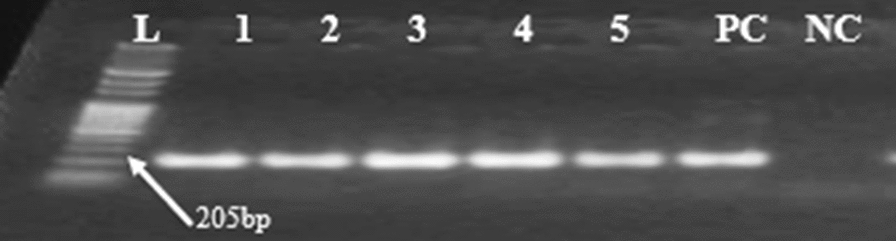
Gel image amplification and detection of *P. falciparum* species using n-PCR, L: 100 bp DNA ladder; L1–L5: samples, PC (L6): positive control (3D7); NC (L7): negative control

Of the 363 samples, 279 (76.8%) were positive for *P. falciparum* by nested PCR amplification (Fig. 2).

#### *Pfmsp-2* genotyping results

**Fig. 3:**
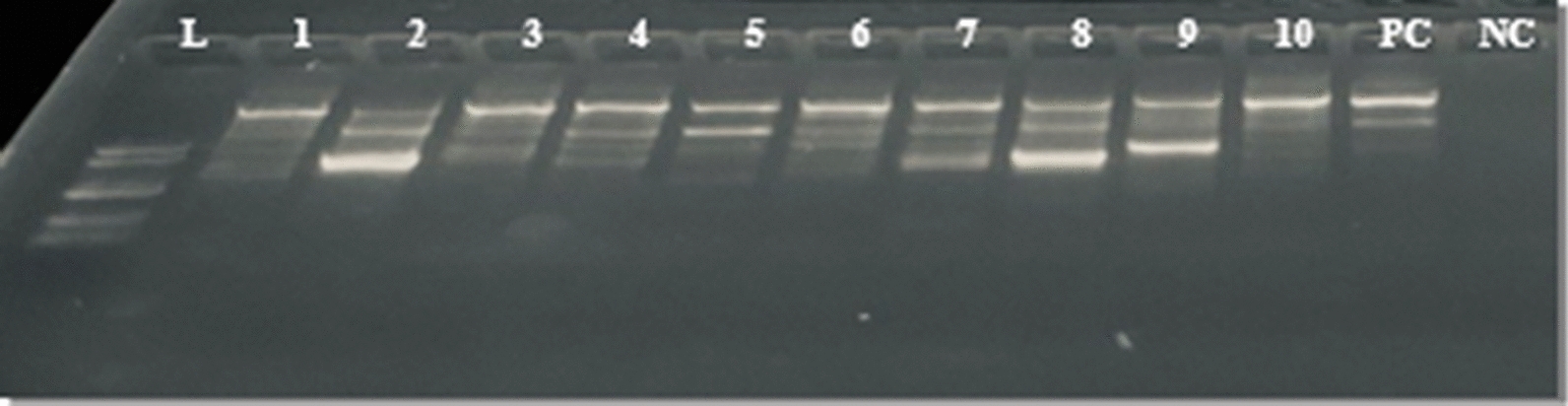
*msp2* amplification gel electrophoresis image, L: DNA ladder 100 bp; L1–10 samples; PC: positive control (3D7); NC: negative control (NC) (NTC); expected bands 350–600 bp

Of the 279 samples positive for *P. falciparum*, 249 (89.2%) were also positive for a single-copy gene called *pfmsp-2*, indicating quality DNA for the subsequent confirmatory analysis of *pfhrp2/3* gene deletions. Thirty of the 279 samples were not amplified for the *msp-2* gene and were excluded from *the pfhrp2/3* genotyping (Fig. 3).

#### Molecular confirmation of *pfhrp2*- and *pfhrp3*-deletions

**Fig. 4:**
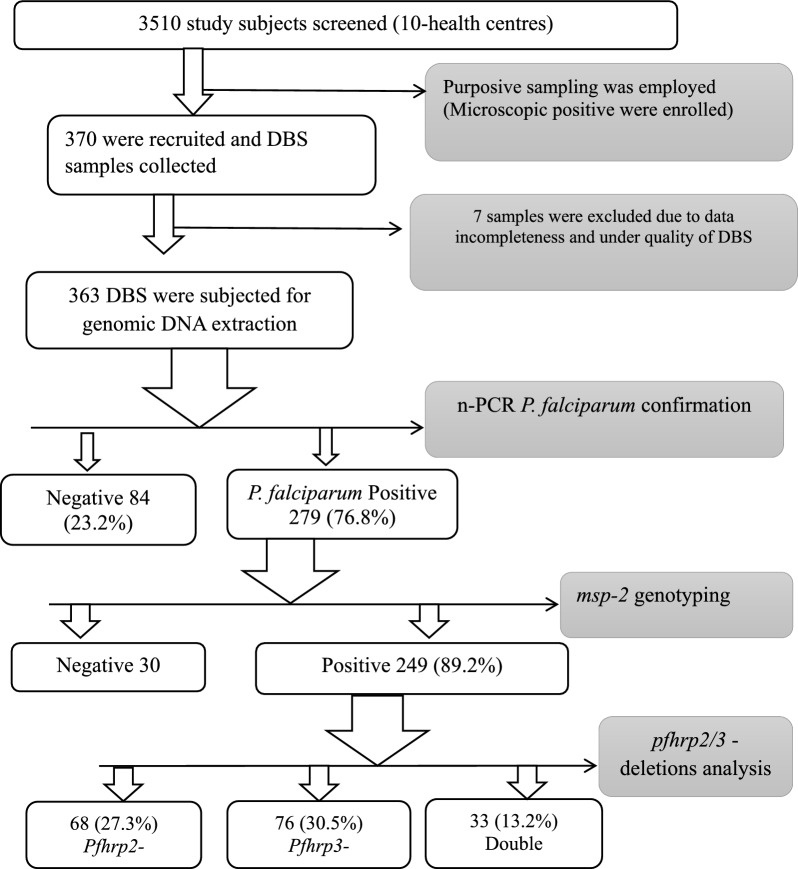
Flow chart illustrates the overall study procedures and results, southern Ethiopia, 2023

Among the 249 *msp-2* positive samples, 144 (57%) showed deletion of at least one of the two genes *hrp2/3*. *hrp2* deletions were observed in 68 (27.3%) of the *P. falciparum* clinical isolates genotyped, while 76 (30.5%) were lacking hrp3 gene. Dual deletion of *hrp2* and *hrp3* genes was observed in 33 (13.3%) of the isolate (Fig. 4).

### Sensitivity of PfHRP-2 RDT against polymerase chain reactions (PCR)

Using PCR as a reference test for detecting *P. falciparum* infections, the sensitivity of the PfHRP-2 SD Bioline RDT test was 76.5% (95% CI 71.42–81.13%), with a kappa coefficient value of 0.613, indicating good agreement between them (Table 3).

**Table 3:** Performance of SD Bioline PfHRP2-RDT compared with the reference PCR test

HRP-2 RDT	PCR			
	+	−		
+	238	73	311	Sensitivity, (95% CI), 76.53 (71.42–81.13) Specificity, (95% CI), 21.15 (11.06–34.70) PPV, (95% CI), 85.3 (83.27–87.13) NPV, (95% CI), 13.10 (7.91–20.90) Accuracy, (95% CI), 68.60 (63.55–73.34) Cohen’s kappa (95% CI) 0.613
−	41	11	52	
N	279	84	363	

+: positive; −: negative, N: total; PPV: positive predictive value; NPV: negative predictive value

### Proportional analysis of *pfhrp2/3* deletions in concordant and discordant sample sets

**Fig. 5:**
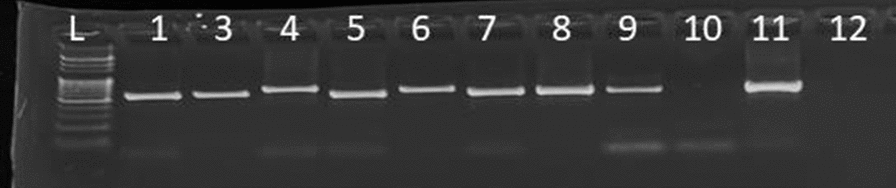
*Pfhrp3–exon-2* amplification results; L: 100 bp DNA ladder; L1–L10: samples, L11: 3D7-positive control; L12: negative control (expected bands = 600–650 bp)

**Fig. 6:**
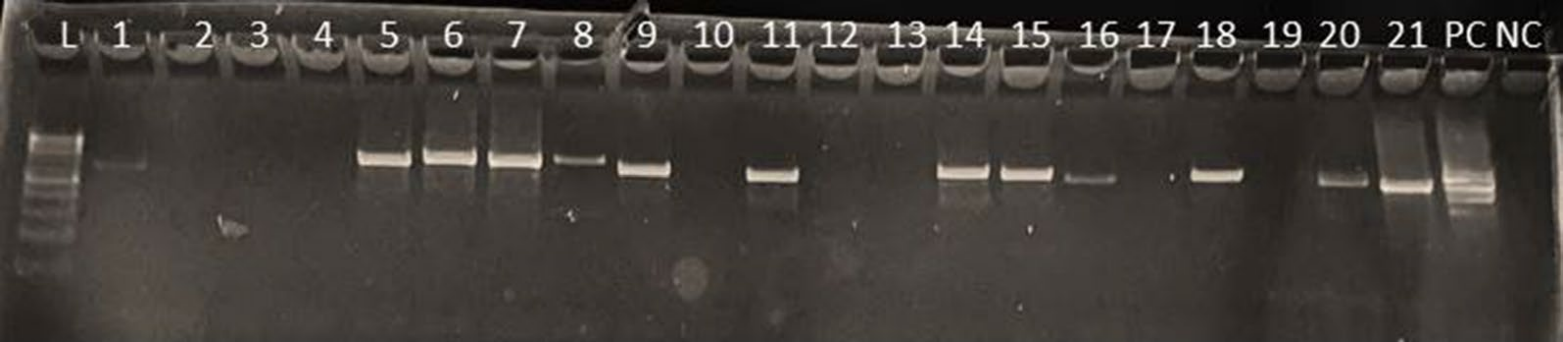
*Pfhrp2–exon-2* amplification results; L: 100 bp DNA ladder; L1–L21: samples; PC: 3D7-positive control; NC: negative control (expected bands = 600–650 bp)

Out of 249 *msp-2* positive samples, 18.8% (47/249) showed discordant results between smeared microscopy and the PfHRP-2 RDT kit. These discordant samples are classified as false-negative RDTs. Conversely, 81.1% (202/249) showed testing agreement between the testing tools. The study found that double *pfhrp2* and *pfhrp3* deletions were absent in all the concordant samples. On the other hand, out of 202 concordant samples, 15.3% (31/202) and 18.3% (37/202) of them, respectively, had *pfhrp2* and *pfhrp3* deletions (Figs. 5, 6).

Among the 47 false negative RDT samples, 70% (33/47) had a dual deletion, whereas 8.5% (4/47) and 12.8% (6/47) had single deletions in the *pfhrp2* and *pfhrp3* genes, respectively. Further, both the *hrp2* and *hrp3* genes were found to be positive in 8.5% (4/47) of the samples that showed discordance. The parasite density in each of these four *hrp2* and *hrp3* positive samples was below 200 parasites per microlitre (μL) of blood.

### Geographical distribution of gene deletions across the study areas

**Fig. 7:**
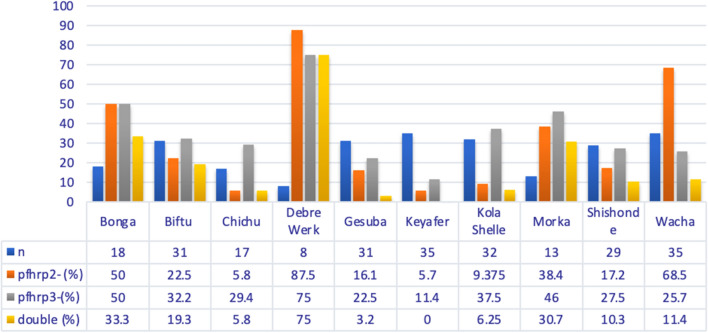
Distribution of single and double *pfhrp2/3*-deletions across the study areas in southern Ethiopia (n-sample size from each site)

The study findings revealed the prevalence of *pfhrp2/3* gene deletion varied across the study sites. Debrewerk, Wacha, and Bonga health centres, 87.5% (7/8), 68.5% (24/35), and 50% (9/18), respectively, exhibited the highest *pfhrp2* gene deletions. Conversely, the highest percentages of *pfhrp3* gene deletions were found in Debrewerk, Bonga, and Morka Health Centres, 75% (6/8), 50% (9/18), and 46.1% (6/13), respectively. No dual deletions were detected at Keyafer Health Centre (Fig. 7).

### Association between *pfhrp2/3* deletions and potential influencing factors

Of the 249 study subjects genotyped for *pfhrp2/3*, 43.5% (156) had a history of previous malaria exposure, and 64.7% (101) had at least one *pfhrp2/3* deletion. Specifically, 23.7% (37/156), 28.2% (44/156), and 12.8% (20/156) of patients showed *pfhrp2, pfhrp3*, and double deletions, respectively. On the contrary, among the study participants with no prior history of malaria exposure 37.3% (93/249), exhibited at least one single or double gene deletion, accounting for 83.8% (78/93). Correspondingly, 33.2% (31/93), 36.5% (34/93), and 14% (13/93) of study participants had *pfhrp2, pfhrp3* and double deletions, respectively. The study found a significant association between previous malaria exposure and gene deletions, with individuals without previous exposure having a higher percentage of deletions (p = 0.015).

Out of 117 individuals with a parasite density of less than 5000/µL of blood, 87 (74.3%) had deletions, either single or double in *pfhrp2/3* genes. Among the 132 individuals with a parasite density greater than 5000/µL of blood, 89 individuals (67.4%) had gene deletions. Overall, the findings indicated that there was no statistically significant association between parasite density and gene deletions (p = 0.314).

## Discussion

The present study determined the molecular epidemiology of *pfhrp2/3* deletions causing false-negative PfHRP2-based rapid diagnosis among symptomatic *P. falciparum* patients in southern Ethiopia. The study is anticipated to be the first to examine whether these gene deletions exist and how common they are in the diverse ecology of southern Ethiopia, as well as a systematic study that follows the WHO gene deletion protocol [19]. The prevalence of *P. falciparum* infection in the study area aligns with findings from a previous report [33].

The current findings revealed that 18.8% (47/249) of samples tested negative for *P. falciparum* when using PfHRP2-RDTs but were found positive through microscopy and PCR analysis. Compared to the 47.8% reported in the Mount Cameroon region [34], the current finding show a smaller discrepancy. Nevertheless, the results obtained thus far surpass the 4.6% false negative rate for RDT that was documented in Central Vietnam [35]. The discrepancies observed in the study imply the possibility of gene deletions, specifically in either the *pfhrp2* or *pfhrp3* gene. Numerous studies conducted worldwide have documented the appearance of *P. falciparum* parasites devoid of these genes, leading to false-negative outcomes in PfHRP-2-based RDTs [22, 23, 34, 35]. False-negative RDTs can have serious consequences, especially when alternative confirmatory tests like microscopy or PCR are unavailable. This situation can lead to delays in anti-malarial treatment, posing a risk to lives, and allowing the potential for ongoing malaria transmission due to escaping diagnostics [23, 31, 36].

Using PCR as the reference test, the sensitivity of the PfHRP2-based RDT was determined to be 76.5%. This figure is lower compared to similar studies conducted in China-Myanmar [36], Ethiopia [37], and French Guiana [38, 39]. However, it is higher than the sensitivity reported in a study conducted in Ethiopia [40]. In Ethiopia, the diagnosis of malaria through microscopy is limited to primary hospitals and health centres [3, 41]. RDTs are used for the majority of malaria diagnoses, especially in peripheral health posts [42], particularly during specific community-driven malaria tests and treatment initiatives. The study findings highlight comprehending the diagnostic accuracy of the most widely used PfHRP2 RDTs in Ethiopia [9] is very crucial to avert catastrophic consequences.

The study found that 27.3%, 30.5%, and 13.2% of *P. falciparum* circulating in southern Ethiopia had *hrp2, hrp3,* and dual *hrp2/3* gene deletions, respectively. Besides, the results revealed the widespread deletions of the *pfhrp2/3* gene in all the health centres that were part of the investigation. The present findings were higher than those of recent studies conducted in Ethiopia [22, 23], which estimated 9.7% and 17.9% among symptomatic *P. falciparum* patients in clinical settings of different regional states, respectively. In contrast to the findings reported by [21], wherein a 100% local prevalence of *pfhrp2* and *pfhrp3* deletions was identified, the current study reveals a comparatively lower prevalence of these deletions. However, the results are comparable with a recent meta-analysis finding conducted on the global prevalence of gene deletions, which were estimated at 21.29%, 34.49%, and 18.65% for single *pfhrp2*, *pfhrp3*, and dual deletions, respectively [43]. The overall prevalence of any *pfhrp2/3–exon-2* deletions in symptomatic *P. falciparum* cases across health facilities was estimated at 144 (57.8%). The finding was higher than studies conducted in Nigeria [44], Rwanda [45], Ghana [46], and Zambia [47], (17%), (23%), (36.2%) (37.5%), respectively. However, the prevalence of deletions was lower than in studies conducted in Sudan [48] and Eritrea [49], which estimated a prevalence of 60% and 62%, respectively. The variations observed could be attributed to differences in transmission intensity, host immune responses, drug regimens, geographical locations, sample sizes, and the laboratory techniques employed to analyse deletions in *pfhrp2/3* genes. Despite these deletions leading to diagnostic challenges, there is compelling evidence suggesting that the *pfhrp2/3* genes are under positive selection within the country [23, 50].

A prior investigation on the consequences of transitioning to PfHRP2-based RDTs indicated a general decrease in the prevalence of mutant *P. falciparum* parasites after the switch to non-HRP2-based RDTs [51]. Conversely, persisting with PfHRP-2 RDTs in regions where mutant *falciparum* parasites contribute to false-negative outcomes led to an increase in their epidemiological impact [52].

Given the comparable amino acid sequences of HRP2 and HRP3 proteins, allowing for mutual recognition [53, 54], the *exon-2*, the main coding regions, was analysed to assess the extent of deletions in both *pfhrp2* and *pfhrp3* genes. This approach, considering potential antigen cross-reactivity, aims to accurately determine the genuine prevalence of gene deletions [24, 55]. The study findings revealed a broader distribution of *pfhrp3* gene deletions compared to *pfhrp2* deletions. These observations align with previous studies conducted earlier [23, 46, 49]. In contrast, studies conducted in Guyana [56], Rwanda [46], Ghana [46], and Peru [17] have revealed that the prevalence of *pfhrp2* is higher than that of *pfhrp3* gene deletions. Furthermore, the current results suggest that the absence of the *pfhrp2* gene may be a more dependable indicator of false-negative outcomes due to its predominant production by parasites [18, 57].

In comparing gene deletion distribution among different health centres, varying proportions of *pfhrp2*- and *pfhp3*-deletions were observed. Double gene deletions (*pfhrp2*- and *pfhrp3*-) were less common. The differences in gene deletion frequencies across study areas may be attributed to variations in the genetic characteristics of local *P. falciparum* populations or variations in malaria transmission intensity at different sites, as previous research has suggested a potential influence of transmission intensity on gene deletion prevalence [25].

The findings indicated a significant association between individuals lacking prior malaria exposure and genetic mutations in *P. falciparum* parasites. Notably, new malaria cases were more frequently associated with parasites exhibiting deleted genes, potentially attributable to the selective pressure exerted by extensive treatment on the parasites [58]. This underscores the necessity for further research to elucidate the impact of these gene mutations on the resilience of parasites and their reaction to pharmaceutical interventions. Broadly speaking, mutations in the *hrp2/3* genes of *P. falciparum* parasites undermine the efficiency of RDTs, elevating the risk of obtaining false-negative results owing to decreased or absent production of the antigen histidine-rich protein [8, 57, 59].

## Study limitations

The research was conducted during the major malaria transmission seasons in Ethiopia, and the investigation did not analyse the occurrence of gene deletions in the parasite population during the minor transmission season. The study presented PCR-based molecular confirmation of *pfhrp2/3* deletions via amplification of the coding region of *exon-2* genes only and did not look at the flanking regions of the genes. Additionally, the molecular detection method employed in this study might underestimate the actual prevalence, as it cannot detect gene-deleted parasites in cases of polyclonal infections.

## Conclusions

In summary, the present investigation highlights the widespread occurrence of *pfhrp2/3* deletions, leading to inaccurate results in PfHRP2-based RDTs, aligning with the WHO recommendations to explore alternative non-HRP2-based RDTs. The study underscores the substantial impact of *hrp2/3* gene deletions in generating false negative test outcomes, thereby jeopardizing the timely detection and treatment of patients.

Therefore, these findings advocate the adoption of non-HRP2-based RDTs as an alternative measure to curb the grave consequences associated with the continued use of these rapid tests, particularly in the studied area and Ethiopia in general.

## Data Availability

All data generated in this study are included in this published article.

## Abbreviations

AAU, ALIPB: Addis Ababa University, Institute of Pathobiology
bp: Base pairs
EPHI: Ethiopian Public Health Institute
FN: False negative
WHO: World Health Organization
*msp-2*: Merozoite surface protein-2
DBS: Dried blood spot
HRP-2-: Histidine-rich protein: 2
*pfhrp-2/3*: *Plasmodium falciparum* histidine-rich protein 2/3
NAAT: Nucleic acid amplification test
NEQAS: National External Quality Assessment Service
PCR: Polymerase chain reaction
TP: True positive
SPSS: Statistical Packages for Social Sciences
